# Supplementary material for: WordCommentsAnalyzer: A windows software tool for qualitative research
Source: F1000Res. 2018 Sep 4;7:536. Originally published 2018 May 3. [Version 2] doi: 10.12688/f1000research.14819.2 (PMC6137411; doi:10.12688/f1000research.14819.2)
Supplement: Supplementary file 3 [file f1000research-7-17614-s0002.tgz › b2a79496-4ada-47c4-9682-a99ce867836d.zip!/Eight Iranian Health Journals/Yafteh _60.docx]

© Yafteh  . This work is licensed under CC BY 4.0 (https://creativecommons.org/licenses/by/4.0/).

Volume & Issue: دوره 6 - شماره 1

Title: ادم پری تومورال همراه مننژیوم ها، نقش رسپتورهای استروئیدی و مشخصات رادیولوژیک

http://yafte.lums.ac.ir/article-1-901-fa.html

مقدمه: علی رغم خصوصیات خوش خیم و اکستراآگزیال مننژیوم ها، این تومورها در بیش از ٦٠% موارد با ادم پری تومورال (PTBE) درنسج مغز اطراف همراه هستند. هدف این مطالعه مشخص کردن نقش میزان مثبت شدن هسته سلول های تومورال از لحاظ رسپتور پروژسترونی (PR) بعنوان فاکتوری از رفتار سلول و پیدا کردن خصوصیات دیگری در MRI و CT بیماران در این رابطه و در مجموع در ارتباط با ادم پری تومورال بوده است.مواد و روشها: در این مطالعه ٧٧ بیمار مبتلا به مننژیوم درون جمجمه در بیمارستان امام خمینی تهران در سال های فاصل 1382-1378 به طور آینده نگر پیگیری شده و برای هر بیمار در ابتدا CT Scan و MRI (T1, T2) انجام و براساس تصاویر موجود، قطر، حجم، محل و شکل لبه تومور و وجود PTBE به همراه شکل و اندازه آن بررسی شده است؛ سپس با یافته های هنگام جراحی در آسیب شناسی و در آخر نتایج حاصل از رنگ آمیزی ایمونوهیستوشیمی (IHC) از لحاظ رسپتورهای استروژنی و پروژسترونی مقایسه گردیده است. نتیجه گیری: در مجموع می توان گفت قرار گرفتن تومورهای مننژیوم اینتراکرانپال در یک درجه و نوع بافت شناسی برای پیش بینی رفتار تومور کافی نبوده و باید اطلاعات بیشتری را در حد سلولی برای پیش بینی رفتار تومور نمود. یافته ها: اشکال مننژیوم با میزان رسپتور پروژسترونی (PR Mod-Class) بالاتر اولاً حجم کوچکتری داشته؛ ثانیاً لبه اطراف تومور به شکل صاف بوده و نمای غیر تهاجمی تری را به نمایش گذاشته، بطوری که فضای آراکنوئید اطراف تومور در بیش از ٥٠% آن قابل مشاهده است و ثالثاً احتمال دیدن ادم پری تومورال کمتر بوده؛ اما در مقابل معمولاً مدت بیماری طولانی تر و خونریزی هنگام عمل کمتر بوده است.

Title: تأثیر دمکرده گل گاو زبان بر بیوشیمی ادرار و خون بیماران با نارسایی مزمن کلیه غیر دیالیزی

http://yafte.lums.ac.ir/article-1-902-fa.html

مقدمه: بیماری نارسایی مزمن کلیه (CRF) از جمله بیماریهایی است که درمان قطعی ندارد. افزایش عملکرد کلیه، کاهش فشار خون و وزن از استراتژی های مهم کنترل و جلوگیری از پیشرفت این بیماری است. گیاه گاو زبان دارای فواید افزایش دفع ادرار، کاهش فشار خون و تقویت عملکرد کلیه است. هدف از انجام این مطالعه، تعیین تأثیر مصرف دمکرده گل گاو زبان بر بیوشیمی ادرار و خون مبتلایان به CRF غیر دیالیزی شهر خرم آباد بود. یافته ها: نتایج نشان داد بعد از مصرف دم کرده گل گاو زبان،حجم ادرار24ساعته، دفع اسید اوریک و پتاسیم ادرار و GFR به طور معنی داری افزایش یافته بود (05/0p<). وزن و فشار خون بیماران نیز به طور محسوسی کاهش یافته بود (05/0p<). گرچه شاخص هایی نظیر نیز مثل کراتی نین، اسید اوریک، سدیم و پتاسیم در سرم خون کاهش یافت اما تفاوت آماری معنی دار نبود. نتیجه گیری: مصرف دمکرده گل گاو زبان بر بیوشیمی ادرار و خون بیماران با نارسایی مزمن تأثیر داشته و باعث کاهش فشار خون و وزن بیماران شده بود. مواد و روشها: در این کارآزمایی بالینی یک گروه 25 نفره از بیماران بالغ مبتلا به CRF به طور تصادفی انتخاب و برای آنها دمکرده گل گاو زبان به مدت یک هفته روزی دو بار هر بار 70 گرم تجویز شد. بار اول قبل از مصرف دمکرده و در شروع مطالعه، بار دوم روز پنجم مصرف و بار سوم یک روز بعد از اتمام مصرف گیاه، از بیماران معاینه، مصاحبه و آزمایش های ادرار و خون به عمل آمد. نتایج هر سه بار بررسی ثبت گردید. اطلاعات مرحله اول به عنوان نتایج قبل از مصرف و میانگین اطلاعات مراحل دوم و سوم به عنوان نتایج بعد از مصرف گیاه محسوب گردید. برای تعیین تأثیر گیاه از آزمون های آماری T و مقایسه زوج ها استفاده شد. همین طور، اختلاف میانگین نتایج هر پارامتر آزمون و تفسیر گردید.

Title: بررسی وضعیت آهن در بیماران مبتلا به آترواسکلروز کرونر

http://yafte.lums.ac.ir/article-1-903-fa.html

مقدمه: در تحقیقات متعدد ثابت شده است که رادیکال های آزاد از طریق اکسیداسیون لیپیدها موجب تغییرLDL، تسهیل رسوب آن و تشکیل پلاک های آترواسکلروتیک می گردند. تولید رادیکال آزاد در حضور آهن،کاتالیز و تشدید می شود. این مطالعه با هدف ارزیابی شاخص های سرمی وضعیت آهن در بیماران مبتلا به بیماری کرونر قلب (CAD)،ارزیابی شده با آنژیوگرافی کرونر در مرکز قلب و عروق شهید مدنی تبریز انجام گردید. مواد و روشها: در این مطالعه 241بیمار(182مرد و59 زن) مراجعه کننده به مرکز قلب برای انجام آنژیوگرافی کرونر، درمحدوده سنی 77-40 سال انتخاب و بررسی شدند. 168 نفر (134 مرد و34 زن) دچار آترواسکلروز کرونر (گروه با CAD) و 73 نفر (48مرد و 25زن) فاقد آن بودند (گروه بدون CAD). دو گروه از نظر سن و جنس همسان بودند.عوامل خطر اصلی CAD شامل سن، جنس، لیپیدهای سرم، چاقی، سیگار، فشارخون، شاخص های سرمی وضعیت آهن (آهن، TIBC،درصد اشباع ترانسفرین و فریتین) وCRPبا روش های استاندارد تعیین شدند. نتیجه گیری: با توجه به پائین بودن در خور توجه میانگین غلظت سرمی TIBCدر مردان باCAD درمقایسه با مردان بدون CAD، چنین استنباط می شود که ذخایر آهن بدن وعروق ممکن است در ایجاد آترواسکلروز کرونر در مردان تحت بررسی نقش داشته باشد. یافته ها: در مردان باCAD مورد بررسی، میانگین غلظتTIBC سرم بطور معنی دار کمترازمردان بدونCAD بود (05/0P<). میانگین غلظت سایر شاخص های سرمی آهن(فریتین، آهن و درصداشباع ترانسفرین) در مردان با CAD، بالاتراز میانگین آنها درمردان بدونCAD بود؛ اما این تفا وت ها معنی دار نبود. در زنان تحت مطالعه در دو گروه میانگین غلظت شاخص های سرمی آهن تفاوت معنی دار ی را نشان نداد. در افراد بررسی شده، بین غلظت تری گلیسرید سرم با فریتین (196/0r=) و آهن سرم (239/0= r) و ارتباط های مثبت و معنی دار دیده شد (01/0p=).

Title: بررسی انواع کلاژن جفت های ایرانی به منظور تعیین میزان استاندارد

http://yafte.lums.ac.ir/article-1-905-fa.html

مقدمه: تغییرات در شکل کلاژن های بافتی همراه با بعضی اختلالات ژنتیکی و متابولیکی گزارش شده است. این تغییرات با اندازه گیری زنجیره های کلاژن از طریق الکتروفورز بر پایه ژل پلی آکریل آمید در حضور سدیم دودسیل سولفات (SDS-PAGE) به طور کمی تخمین پذیر است. این مطالعه به منظور یافتن ارتباط بین کلاژن های جفت انسانی طبیعی و ترم ایرانی و تعیین میزان استاندارد برای کلاژن های جفتی میزان کلاژن، توتال پروتئین، درصد زنجیره های کلاژن ها و حرکت الکتروفورتیکی صورت گرفته است نتیجه گیری: نتایج حاصل از مطالعه نشان داد که میزان زنجیره α1 کلاژن نوع III حدود 23درصد کلاژن تام است. یافته ها: در این بررسی معلوم شد بین میزان کلاژن نوع IV جفت و پروتئین توتال، مقدار ضریب همبستگی 0.993) = (r و بین وزن جفت بعد از شستشوی کامل با میزان کلاژن نوع I + III ضریب همبستگی 0.77) (r = دارد . مواد و روشها: بدین منظور برای استخراج کلاژن نوع IV تعداد پنج جفت طبیعی و ترم (38 هفته ای) فقط یک بار و برای استخراج کلاژن نوع I+III تعداد پنج جفت طبیعی و ترم (38 هفته ای) دو بار تحت هضم آنزیمی قرار گرفتند. مراحل استخراج کلاژن ها با الکتروفورز بر پایه ژل پلی آکریل آمید در حضور سدیم دو دسیل سولفات کنترل شد.

Title: نقش مشارکت مادران در تغییر عملکرد آنان در زمینه رشد و تغذیه کودک در شهرستان اصفهان در سال 1382

http://yafte.lums.ac.ir/article-1-906-fa.html

مقدمه: بررسی کشوری رشد کودکان نشان داده است که درصد زیادی از کودکان کشور دچار افت رشد هستند. از طرفی، مشارکت مردمی از اصولی است که سازمان بهداشت جهانی و منابع علمی برای ارتقای سلامت بر آن تاکید دارند. بنابراین می توان یکی از راهکارهای بهبود رشد کودکان را توجه به مشارکت بیشتر مادران دانست. در مطالعه حاضر با ارائه یک الگوی مشارکت مادران، بهبود عملکرد آنان در مراقبت از کودکان خود بررسی می شود. مواد و روشها: مطالعه حاضر از نوع کارآزمایی مبتنی بر جامعه (community - based trial) است و بر روی 74 زوج مادر و کودک انجام شده است. بدین منظور الگویی با استفاده از سه منبع (سطوح مشارکت مردمی، تجارب کشور ایران و سایر کشورها در مورد مشارکت و الگوی CIPP) و هم چنین در نظر گرفتن ویژگی های فرهنگی – اجتماعی و نظام ارائه خدمات بهداشتی کشور طراحی شد. موضوع مشارکت، رشد و تغذیه کودکان بود. برای اجرا با همراهی گروه اصلی مادران داوطلب، نیازسنجی، برنامه ریزی،‌اجرا، نظارت و ارزشیابی پروژه انجام شد. عملیات با آموزش گروه یاد شده آغاز و توسط آنها به کلیه مادران انتقال داده شد. به منظور ارزشیابی این الگو، شاخص های دستیابی به اهداف در زمینه دانش و عملکرد و توانمندی های فردی و اجتماعی مادران تعیین گردید و از طریق مصاحبه، مشاهده و تکمیل پرسش نامه، اطلاعات جمع آوری و با نرم افزار SPSS و آزمون های آماری ویل کاکسون، مک نمار و t زوج تحلیل گردید. یافته ها: میانگین مجموع نمرات آگاهی مادران در زمینه محتوای آموزشی الگو، قبل و پس از مداخله به ترتیب 23 و 34 و میانگین مجموع نمرات عملکرد آنان قبل و پس از مداخله 3/47 و 2/60 بود (اختلاف معنی دار با 001/0p<). شاخص های حاصل از مشارکت مانند تأثیر بر اعتماد به نفس، اشتیاق به یادگیری مطالب جدید، تمایل به استمرار فعالیت گروه، تمایل به حل مشکلات و ابتکار در زندگی تغییرات شایان توجهی داشت. نتایج طرح از جمله اثربخشی، حاصل نهایی، استمرار برنامه و قابلیت انتقال آن با الگوی CIPP ارزشیابی شد.نتیجه گیری: افزایش سطوح همکاری مادران در ابعاد مختلف مشارکت شامل ارزیابی نیازها،‌اجرا، نظارت، ارزشیابی و تشکیل یک تشکل مردمی (NGO) بهبود قابل توجهی را در دانش و عملکرد مادران در زمینه مراقبت ازکودکان خود ایجاد کرد. هم چنین سبب افزایش توانمندی های فردی و اجتماعی آنان شد که بیانگر کارایی الگوی ارائه شده بود. از آنجا که در طراحی این الگو به ویژگی های فرهنگی ـ اجتماعی و نظام ارائه خدمات بهداشتی کشور توجه شده است، می توان آن را در سایر مناطق و برای توسعه خدمات بهداشتی دیگر به کار برد.

Title: صلاحیت بینایی رانندگان اتوبوس های بین شهری تهران در مقایسه با استانداردهای ایران و اروپا

http://yafte.lums.ac.ir/article-1-907-fa.html

مقدمه: با افزایش تعداد وسایل نقلیه جاده‌ای، به کارگیری رانندگانی که از مهارتهای حسی و حرکتی بیشتری دارند برای حصول ایمنی ترافیکی اجتناب ناپذیر است. بینایی یکی از حواسی است که به طور مطلق برای رانندگی ایمن لازم است و تقریباً 95% از ورودی های حسی مغز که برای رانندگی لازم است متعلق به حس بینایی است. به همین منظور در کشورهای مختلف استانداردهایی مقرر شده تا در هنگام صدور و تمدید گواهینامه،سلامت بینایی رانندگان با این استانداردها مطابقت داده شود. هدف از انجام این مطالعه بررسی وضعیت دید رانندگان اتوبوس های بین شهری تهران و مقایسه آن با استانداردهای چهار کشور ایران، انگلستان، فرانسه و فنلاند بود. مواد و روشها: ابتدا تعداد312 نفر از رانندگان شاغل در پایانه های جنوب، شرق، غرب و بیهقی تهران با روش نمونه گیری تصادفی ساده انتخاب شدند. سپس پرسش نامه ای شامل مشخصات فردی و اطلاعات شغلی رانندگان با روش مصاحبه حضوری برای هر یک از شرکت کنندگان تکمیل گردید. سپس معاینه حدت و میدان بینایی با استفاده از چارت اسنلن و آزمون رویارویی (Confrontation) انجام شد و اطلاعات به دست آمده با نرم افزار SPSS و آزمون های آماری مربوط تحلیل گردید. نتیجه گیری: بر اساس یافته های به دست آمده لازم است که با یک بررسی اصولی و علمی استانداردهای قابل قبولی را برای صدور انواع گواهی نامه تدوین کرد. یافته ها: متوسط سن افراد مطالعه شده 78/42 سال و متوسط مدت زمان اشتغال به کار رانندگی آنها 15/18 سال بود.به طور متوسط 19/23 ماه (حدود 2 سال) از آخرین معاینه چشمی آنها گذشته بود که بیشترین علت آن معاینه به هنگام تعویض دفترچه مدت کار و بازرسی سرعت بود. 28/51% افراد بهترین حدت بینایی در حد 10/10 و 71/48% افراد بهترین میدان دید در حد 180 درجه نصف‌النهار افقی داشتند. در مقایسه‌ دید افراد مورد مطالعه با استانداردهای بینایی رانندگی در ایران 6/11% واجد صلاحیت رانندگی نبودند. هم چنین 7/7% با قوانین انگلستان، 7/49% با قوانین فرانسه و 1/17% با قوانین فنلاند واجد استانداردهای بینایی رانندگی نبودند. میانگین سن افراد واجد صلاحیت بینایی برای رانندگی24/42 سال و میانگین سن افراد فاقد صلاحیت بینایی برای رانندگی 88/46 سال بود که با استفاده از آزمون آماری T این اختلاف با 009/0=P از نظر آماری معنی‌دار بود. با استفاده از آزمون آماری Kruskal-Wallis مشخص شد که میزان حدت بینایی در گروه سنی بالای 50 سال کمتر از گروه های سنی 20 تا 50 سال و زیر 20 سال است و این اختلاف با 001/0 P< از نظر آماری معنی دار بود، اختلاف میدان بینایی بین گروه های سنی معنی‌دار نبود.

Title: تأثیرآنتی اسپرم آنتی بادی های اندازه گیری شده به روش واکنش آگلوتیناسیون مختلط مستقیم بر درصد لقاح آزمایشگاهی

http://yafte.lums.ac.ir/article-1-908-fa.html

مقدمه: اگر چه لقاح آزمایشگاهی (IVF) برای برطرف نمودن اثر مهاری سطوح بالای آنتی اسپرم آنتی بادی ها بر حرکت اسپرم ابداع شده است؛ ولی در این روش میزان باروری تقریباً در 40% موارد کاهش می یابد. در این مطالعه ارتباط بین آنتی اسپرم آنتی بادیهای اندازه گیری شده به روش واکنش آگلوتیناسیون مختلط (MAR) مستقیم و درصد لقاح در زوجهای نابارور کاندید IVF بررسی شد. مواد و روشها: نمونه های سمن از مردان 80 زوج ناباور کاندید IVF مراجعه کننده به مراکز باروری و ناباروری اصفهان به دست آمد. زوج ها بر اساس درصد لقاح به دو گروه دارای درصد لقاح بالا و درصد لقاح پایین تقسیم شدند. 52 زوج درصد لقاح بالا (65%) و 28 زوج درصد لقاح پایین (35%) داشتند. سپس درصد آنتی اسپرم آنتی بادی ها از کلاس IgA وIgG در نمونه های سمن به روش MAR مستقیم تعیین شدند. تجزیه و تحلیل آماری با استفاده از آزمون های X2، T مستقل و همبستگی پیرسون انجام گرفت. نتیجه گیری: نتایج این مطالعه به وضوح نشان می دهد که سطوح بالای آنتی اسپرم آنتی بادی درصد لقاح را کاهش می دهد. بنابراین پیشنهاد می گردد که زوجها با سطوح بالای آنتی اسپرم آنتی بادی ها، کاندید تزریق داخل سیتوپلاسمی اسپرم (ICSI) گردند. یافته ها: میانگین درصد آنتی اسپرم آنتی بادی ها در گروه های بادرصد لقاح بالا و پایین اختلاف معنی داری داشت (001/0p <). از نظر آماری ارتباط معکوس معنی داری بین درصد لقاح و سطح آنتی اسپرم آنتی بادی ها از کلاس IgA مشاهده شد (001/0p< و 56/0r=). همچنین از نظر آماری ارتباط معکوس معنی داری بین درصد لقاح و سطح آنتی بادی از کلاس IgG مشاهده شد (001/0p< و 42/0-r=).

Title: ویژگی ها و درجه های مختلف تومورهای اولیه مغزی در بیماران بستری شده در بیمارستان الزهرای اصفهان بر اساس طبقه بندی WHO (1379-1375)

http://yafte.lums.ac.ir/article-1-909-fa.html

مقدمه: حدود نیمی از تومورهای مغزی اولیه و بقیه متاستاتیک هستند. تومورهای سیستم عصبی خصوصیات منحصر بفردی دارند که آنها را از نئوپلاسم های سایر نواحی بدن متمایز می سازد. سازمان بهداشت جهانی تومورهای سیستم عصبی مرکزی را براساس میزان بدخیمی به درجه های 1 تا 4 طبقه‌بندی نموده است. هدف از انجام این مطالعه بررسی توزیع سنی ـ جنسی و محل آنامیک و درجه های مختلف تومورهای اولیه بر اساس طبقه بندی WHO بود. نتیجه گیری: برخلاف نتایج به دست آمده در مورد توزیع جنسی تومورهای اولیه مغزی در این مطالعه، در سایر مطالعات ابتلای مردان به این تومورها شایع تر از زنان گزارش شده است. به علاوه در منابع موجود گلیوما به عنوان شایع ترین تومور اولیه مغزی ذکر شده درحالی که در این بررسی مننژیوم فراوان ترین تومور مشاهده شده بوده است. یافته ها: اختلاف معنی‌داری بین فراوانی نسبی تومورهای اولیه مغزی در دو جنس مشاهده نشد. اکثریت مبتلایان بیش از 35 سال داشتند. شایعترین محل آناتومیک گرفتاری، نیمکره‌ های مغزی و شایع ترین نوع تومور مننژیوم بود. اکثریت تومورها در درجه 1 درجه بندی سازمان بهداشت جهانی قرار داشتند. مواد و روشها: این مطالعه روی 400 بیمار مبتلا به تومورهای اولیه مغزی بستری شده در بیمارستان الزهرای اصفهان در یک دوره پنج ساله صورت گرفته است. اطلاعات مربوط به سن، جنس و محل آناتومیک از پرونده بیماران استخراج شد. نوع آسیب شناختی هر ضایعه براساس مطالعه ماکروسکوپی و میکروسکوپی نمونه جراحی شده تعیین گردیده و بر پایه تشخیص، درجه هریک از نمونه‌ها طبق درجه بندی WHO مشخص شده است.

Title: تعیین سطح آندروژن در بیماران افسرده سنین باروری مراجعه کننده به درمانگاه های اعصاب و روان شهرستان خرم آباد

http://yafte.lums.ac.ir/article-1-910-fa.html

مقدمه: افسردگی یکی از شایعترین اختلالات روان پزشکی است که شیوع آن در زنان دو برابر بیشتر از مردان است.تستوسترون سرم را با بروز اختلالات رفتاری در زنان و مردان مطرح می نماید. هدف از انجام این مطالعه تعیین سطح آندروژن به خصوص تستوسترون در بیماران افسرده سنین باروری بود. نتیجه گیری: مطالعه نشان می دهد آندروژن به خصوص تستوسترون نقش مهمی در پاتوژنز افسردگی دارد. یافته ها: نتایج نشان داد که بین سطح سرمی تستوسترون توتال (ng/dl 4/0 و ng/dl 3/202) و آزاد (ng/dl 1/0 و ng/dl 3/140) در گروه مورد نسبت به گروه شاهد اختلاف معنی داری وجود داشت (05/0p<)؛ در حالی که بین سطح سرمی DHEAS در دو گروه اختلاف معنی داری وجود نداشت. مواد و روشها: پژوهش حاضر با هدف تعیین سطح آندروژن در بیماران افسرده در سنین باروری و مقایسه آن با افراد سالم در سال 1381 انجام شد. پژوهش از نوع case - control بوده که در آن 30 نفر از خانم های سنین باروری در گروه های مورد و 30 نفر در گروه شاهد از نظر تستوسترون، دهیدرو اپی آندروسترون سولفات، مورد بررسی قرار گرفتند. این افراد از نظر سن، وزن، شاخص توده بدنی و سیکل قاعدگی کاملا“ همگن بودند. از افراد واجد شرایط نمونه گیری به عمل آمد و میزان تستوسترون در آزمایشگاه به روش RIA (رادیوایمنواسی) اندازه گیری شد.

Title: بررسی بیماری قلبی درافراد بتا تالاسمی ماژور مراجعه کننده به بیمارستان شهید مدنی خرم آباد درنیمه اول سال 1382

http://yafte.lums.ac.ir/article-1-911-fa.html

مقدمه: بیماران تالاسمی ماژور به علت آنمی شدید به طور مداوم به تزریق خون نیاز دارند. یکی از عوارض مهم در این بیماران رسوب آهن در بافت قلب است که باعث فیبروز و اختلال کار قلب می شود. اختلال قلبی یکی از علل اصلی مرگ و میر در این بیماران است. هدف ازاین مطالعه بررسی عوارض قلبی دربیماران بتا تالاسمی ماژور بود. مواد و روشها: این مطالعه به صورت توصیفی ـ تحلیلی انجام گرفت. حجم نمونه کلیه بیماران بتا تالاسمی ماژور مراجعه کننده به بیمارستان شهید مدنی خرم آباد در نیمه اول سال 1382 (100 بیمار) را شامل می شد. متغیرهای این مطالعه شامل سن, جنس, فریتین سرم, سن تزریق خون, سن استفاده از دسفرال, گروه خونی و سطح هموگلوبین بود. ازتمام بیماران معاینه کامل واکوکاردیوگرافی توسط متخصص قلب انجام و اطلاعات با پرسش نامه جمع آوری شد و به وسیله برنامه SPSS تحلیل آماری انجام گرفت. نتیجه گیری: بر اساس آزمون آماری کای ـ دو بین سن بیماران، سن استفاده از دسفرال و فریتین سرم با بیماری قلبی رابطه معنی دار وجود داشت (05/0p<)؛ اما بین جنس بیماران و گروه خونی رابطه معنی دار پیدا نشد. پیشنهاد می شود که تزریق خون و استفاده از دسفرال به طور منظم در بیماران بتاتالاسمی ماژور انجام شود. همچنین بررسی های دوره ای کار قلب در این بیماران ضروری است. یافته ها: از 100 بیمار مورد مطالعه 33 درصد از بیماران دارای بیماری قلبی بودند. از جمله 6 نفر اختلال سیستولیک بطن چپ از نوع محدود کننده واختلال دیاستولیک بطن چپ باکسر جهشی 55-50 درصد داشتند. بزرگی هر چهار حفره و کاردیو میوپاتی بانارسایی برون ده بالا باکسرجهشی 50-45 درصد در 2 بیمار مشاهده شد. فشار خون ریوی شدید ,نارسایی دریچه آئورت و سه لختی, اختلال بطن چپ و راست, پریکاردیال افیوژن باکسرجهشی 55-45 درصد در 2 بیمار و بزرگی بطن چپ به تنهایی یا همراه بزرگی بطن راست باکسرجهشی 60 درصد در بقیه بیماران قلبی وجود داشت. 5/54% بیماران قلبی مذکر و 5/45 % مونث بودند. در 23% فریتین سرم بین 2000-1501 نانوگرم درمیلی لیتر بود. بیشترین تعداد بیماران درگروه سنی 20-11 سال قرار داشتند. سن شروع تزریق خون در20%بین یک تا دوسالگی بوده است. سن استفاده از دسفرال در 25% بعد از پنج سالگی بود. بیشتر بیماران تالاسمی مبتلا به بیماری قلبی، گروه خونی O داشتند. دو مورد مرگ به علت نارسایی قلبی وجود داشت.

Title: عفونت منتشر به دنبال واکسیناسیون ب.ث.ژ در شیر خوار 8 ماهه

http://yafte.lums.ac.ir/article-1-912-fa.html

مقدمه: عفونت منتشر باسیل کالمت ـ گرین (ب.ث.ژ ) متعاقب تلقیح واکسن زنده بیشتر به عنوان تظاهری از نقص ایمنی در کودکان در نظر گرفته می شود. ولی در نیمی از موارد که به عنوان موارد ایدیوپاتیک تعریف می شود هیچ گونه نقص ایمنی شناخته شده ای وجود ندارد. نتیجه گیری: گرچه واکسن ب.ث.ژ از سال 1921 مورد استفاده می گردد و اثرات آن در پیشگیری از سل منتشر، مننژیال و سل ریوی به اثبات رسیده است ولی اختلاف نظر در مورد این واکسن همچنان ادامه دارد. خطیرترین عارضه واکسن ب.ث.ژ عفونت منتشر است که ممکن است منجر به مرگ بیمار شود. معرفی بیمار: شیر خوار 8 ماهه پسر که با مشکل وجود زخم در محل واکسیناسیون (ب.ث.ژ ) که از پنج ماه قبل ایجاد شده بود، بستری گردید. وی هم چنین از دو هفته قبل دچار تب، سرفه های خلط دار و اختلال تنفسی شده بود. بیمار تحت درمان آنتی بیوتیکی وسیع الطیف قرار گرفت ولی پاسخ درمانی مناسبی نداشت. در معاینات بالینی بعدی توده ای به ابعاد 3×3 در(Left Upper Quadrant) LUQ لمس می شد. در عکس قفسه سینه تصویر رتیکولر منتشر و خوردگی در دنده هفتم مشهود بود. بیمار به مدت دوازده روز بستری بود و در نهایت فوت نمود. در کالبد شکافی بیمار در تمام نمونه های برداشته شده از اعضای مختلف تجمع فراوانی از باسیل اسید فاست وجود داشت و تشخیص نهایی عفونت منتشر سلی (BCGOSIS) بود.
